# Cell cycle regulation in NAFLD: when imbalanced metabolism limits cell division

**DOI:** 10.1007/s12072-020-10066-6

**Published:** 2020-06-22

**Authors:** Matias J. Caldez, Mikael Bjorklund, Philipp Kaldis

**Affiliations:** 1 WPI Immunology Frontiers Research Centre, Osaka University, 3-1 Yamadaoka, Suita, Osaka 565-0871 Japan; 2 Zhejiang University-University of Edinburgh (ZJU-UoE) Institute and 2nd Affiliated Hospital, Zhejiang University School of Medicine, 718 East Haizhou Rd., Haining, 314400 Zhejiang People’s Republic of China; 3 Department of Clinical Sciences, Clinical Research Centre (CRC), Lund University, Box 50332, 202 13 Malmö, Sweden

**Keywords:** Liver regeneration, Cell signaling, Metabolism, Cell cycle, NAFLD, Oxidation, Biosynthesis, Lipids, NASH, Senescence

## Abstract

Cell division is essential for organismal growth and tissue homeostasis. It is exceptionally significant in tissues chronically exposed to intrinsic and external damage, like the liver. After decades of studying the regulation of cell cycle by extracellular signals, there are still gaps in our knowledge on how these two interact with metabolic pathways in vivo. Studying the cross-talk of these pathways has direct clinical implications as defects in cell division, signaling pathways, and metabolic homeostasis are frequently observed in liver diseases. In this review, we will focus on recent reports which describe various functions of cell cycle regulators in hepatic homeostasis. We will describe the interplay between the cell cycle and metabolism during liver regeneration after acute and chronic damage. We will focus our attention on non-alcoholic fatty liver disease, especially non-alcoholic steatohepatitis. The global incidence of non-alcoholic fatty liver disease is increasing exponentially. Therefore, understanding the interplay between cell cycle regulators and metabolism may lead to the discovery of novel therapeutic targets amenable to intervention.

## Introduction

**Fig. 1:**
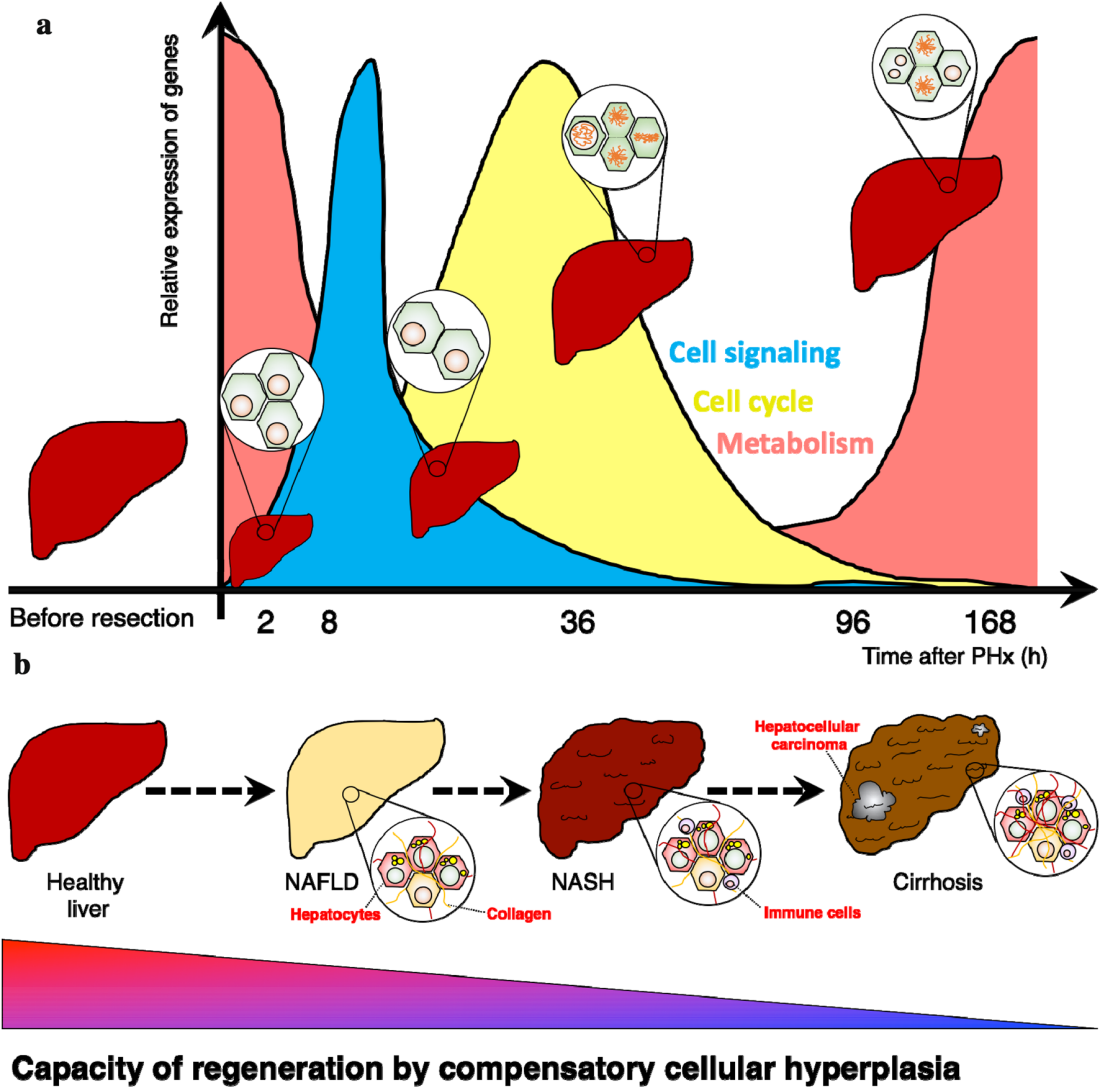
Principles of liver disease, injury, and regeneration. Currently, liver resection and transplantation are the first line therapy offered to patients with a range of liver diseases. **a** Diseased livers display impaired capacity of regeneration after resection. Liver regeneration is a mechanism by which hepatic cells compensate for the loss of liver mass. It is well synchronized, and in a relatively short period of time, the liver returns to its normal size by proliferation of hepatic cells. **b** One of the most common diseases in the clinical setting displays a significant increase in accumulation of lipids in the parenchyma of hepatocytes and is known as non-alcoholic fatty liver disease (NAFLD). NAFLD leads to chronic damage of the hepatic parenchyma, promoting cycles of parenchyma damage and regeneration, leading to fibrosis and activation of immune cells recruited to clear debris. When cycles of damage/regeneration continue in the long-term, there will be more fibrosis and increased inflammation leading to non-alcoholic steatohepatitis (NASH). NASH may evolve to liver cirrhosis, cancer, and end-stage liver disease. During the development of NAFLD and progression to NASH, the capacity of regeneration of the liver by compensatory cellular hyperplasia is dramatically reduced as shown in the colored triangle in which red implies high and blue low capacity of regeneration

The liver is the largest solid organ in the mammalian body. It fulfills essential roles in the gastrointestinal tract, controlling metabolism, detoxification, digestion, and many other essential processes [1]. Due to its functions and the strategic location alongside the gastrointestinal tract, it is constantly exposed to intrinsic and external damage. To maintain functionality, the liver has retained an impressive capacity to regenerate after injury [2]. The mechanisms underlying liver regeneration are classified into acute or chronic damage depending on nature, timing, and duration of the injury [3]. The critical difference is that regeneration after acute damage represents a synchronized physiological process [4]. In contrast, chronic damage is characterized by cycles of damage-regeneration similar to a wound healing response (Fig. 1). In this review, we will explore the interaction between metabolism and cell cycle in tissue regeneration after partial hepatectomy as a model of acute damage. Next, we will compare these processes in chronic damage, focusing especially in non-alcoholic fatty liver disease (NAFLD).

NAFLD represents the most frequent chronic liver disease in the world. It is characterized by the accumulation of lipid droplets in hepatic cells [5], combined with dysregulation of metabolism and cellular division [4]. Many questions remain open about the role of cell cycle and metabolic pathways during hepatic regeneration after acute and chronic damage. To resolve some of these questions, this review is divided into two parts: (1) in the first section, we will summarize recent data on various roles of cell cycle genes involved in hepatocyte division. Simultaneously, we will describe in detail the functions of metabolic genes during liver regeneration after hepatectomy as an example of acute liver damage (Fig. 1a). Then, (2) we will briefly outline data of studies on the regulation of the cell cycle in murine models of NAFLD as an example of chronic damage. Ultimately, we will propose potential nodes for targeting the interface between the cell cycle and metabolism to improve NAFLD treatment. Liver regeneration after hepatectomy is the standard method to study the synchronized regulation of cell cycle and metabolism in vivo. However, it is essential to highlight that the mechanism of tissue regeneration after resection is different from that of NAFLD.

## The regulation of the cell cycle, signaling, and metabolism during acute hepatic damage and regeneration

Liver regeneration after acute injury strands for an adaptive response by which the liver compensates for the loss of functional tissue using cellular hypertrophy (increase in the size of hepatic cells) and/or hyperplasia ([2, 3], Fig. 1a). Two features render liver regeneration unique compared to repair and healing of other tissues. First, before injury, hepatocytes are in a quiescent state. However, when the liver is confronted with damage hepatocytes are able to re-enter the cell cycle despite being fully differentiated. Therefore, they may function as “facultative” stem cells (for more details on the role of liver stem cells in liver regeneration refer to [2]). Second, hepatic regeneration is well synchronized. Third, the rate of cellular division during regeneration is controlled by several factors. Growth factors from other organs including cytokines from an inflammatory response, metabolic signals, circadian rhythm, and blood pressure are among the most significant ones [2].

The best-described models to study liver regeneration after acute damage are (1) liver resection (partial hepatectomy [3]) and (2) toxicological damage [6]. The essential difference is that the latter induces parenchymatic necrosis, and hepatocyte proliferation is integrated into a complex wound healing response with fibrosis and inflammation [6]. In contrast, 2/3 liver resection activates cell division in the absence of any cellular lesion [3]. In the following discussion, the regenerative response of the liver to partial hepatectomy will be divided into three groups of processes, namely: (1) the activation of cellular signaling pathways; (2) regulation of cell cycle progression; and (3) remodeling of metabolism (Fig. 1a). While these events are occurring simultaneously, the proposed classification solely aims to facilitate the description. We will initially introduce general ideas about the regulation of these three processes independently. Subsequently, we will highlight potential nodes of interaction between the three groups of biological processes.

### Regulation of cellular signaling during liver regeneration

**Fig. 2:**
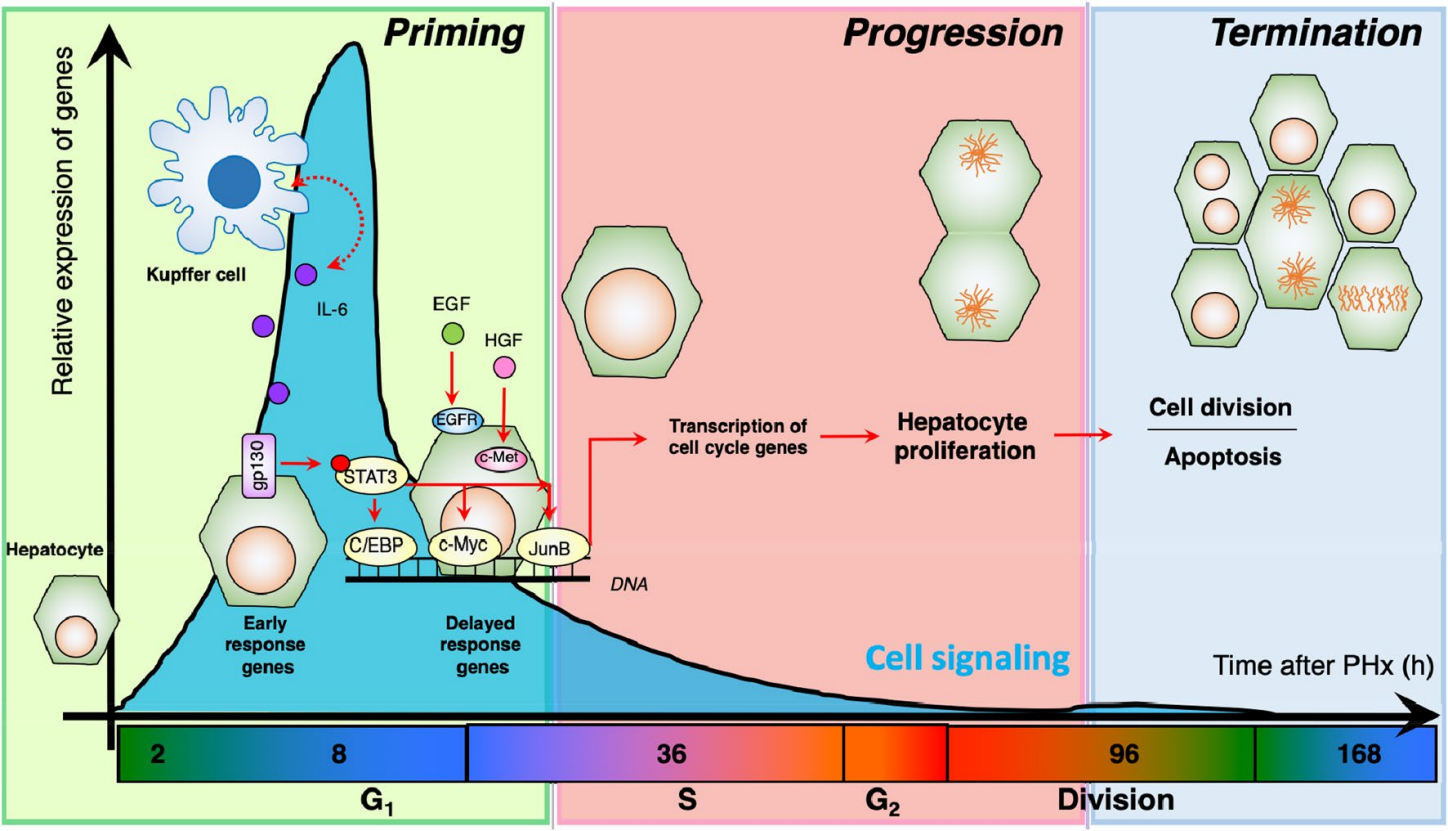
Regulation of cell cycle by auto and paracrine signaling during liver regeneration. Liver regeneration is a well synchronized process by which the liver recovers in response to acute damage. In the most studied model of liver regeneration, 2/3 of the liver mass is removed which triggers a cascade of activation of signaling pathways within hepatocytes promoting regeneration. The first wave of genes is activated by autocrine and paracrine signals. These transcription factors are known as *early response genes* of liver regeneration which license the expression of cell cycle genes during the initiation phase of the regenerative process. The second wave of transcription factors, known as *delayed response genes* of liver regeneration, further promotes cell cycle progression allowing hepatocytes to enter the S-phase of cell cycle. DNA replication is one of the only cellular autonomous processes in hepatocytes, and it always occurs between 32 and 38 h after liver resection, and it is marked by a peak in expression of cell cycle genes, like CDKs and cyclins. After one or two rounds of cell division, most genes that promote cellular proliferation will be reduced in expression and the regenerative process enters the termination phase. The last phase is characterized by a balance of cell division and apoptosis, until the liver reaches the optimal size

Resection of 2/3 of the liver activates an acute phase response followed by a profound rearrangement of the entire cellular signaling network [7]. Based on the consecutive activation of a diverse group of transcription factors (TFs), the process of liver regeneration is conceptually divided into three phases: priming, progression, and termination (Fig. 2). The initiation phase is characterized by a fast activation of early-response genes [2] with a dramatic increase in blood pressure [8]. Collectively, these initiating events have been associated with the release of IL-6 and activation of the IL-6-gp130-Stat3 axis in Kupffer cells [9]. IL-6 from Kupffer cells activates the IL-6 receptor gp130 in hepatocytes which in turn will phosphorylate STAT3, causing increases in transcriptional activity (Fig. 2). Early response TFs promote the activation of a second group known as late-response genes that undergo de novo transcription and translation [2]. Combined with other TFs activated by growth factors like hepatocyte and epidermal growth factors (EGF, HGF), the IL-6-gp130 axis activates expression of key cell cycle genes [10–15].

Cell cycle genes drive the progression phase until the onset of the termination phase [16]. Within 1–2 weeks after resection, any remaining cellular division will eventually cease in murine livers. As such, two weeks after resection, the liver will adjust to the initial pre-resection size and function while in other mammals the process may take longer time. The final stage of regeneration is characterized by the termination phase. Here, the liver-to-body weight ratio is carefully adjusted. The ratio is regulated by the rate of hepatic division and apoptosis until the liver reaches an adequate size and all normal physiological functions are restored [17, 18].

### Regulation of the cell cycle: the role of hypertrophy and cellular division during regeneration

Another distinctive feature of liver regeneration is the dynamic alterations in cellular hypertrophy and division [19]. Normally, after hepatocytes are primed by injury, they temporarily increase in size with a subsequent activation of cell cycle genes and cellular division [17]. However, we and others have shown that an increase in hepatocyte size alone is sufficient to recover organ mass in the absence of cell division in mice. For example deletion of the cell cycle genes Skp2 or Cdk1 [20–23] does not impair regeneration. In addition, early observations indicated that components of the mTOR-AKT signaling are essential to activate an alternative regenerative response to promote tissue recovery by compensatory cellular hypertrophy [19, 24, 25]. Nevertheless, we also observed that compensatory cellular hypertrophy is sufficient for mass recovery, but liver functions are reduced [26]. On top of that, our recent unpublished data suggest that in the long-term, increases in the size of hepatocytes may induce activation of an inflammatory response and fibrosis. In conclusion, in the short term cell hypertrophy may offer an alternative form of tissue regeneration, but may not be sufficient for recovery of liver functions.

**Fig. 3:**
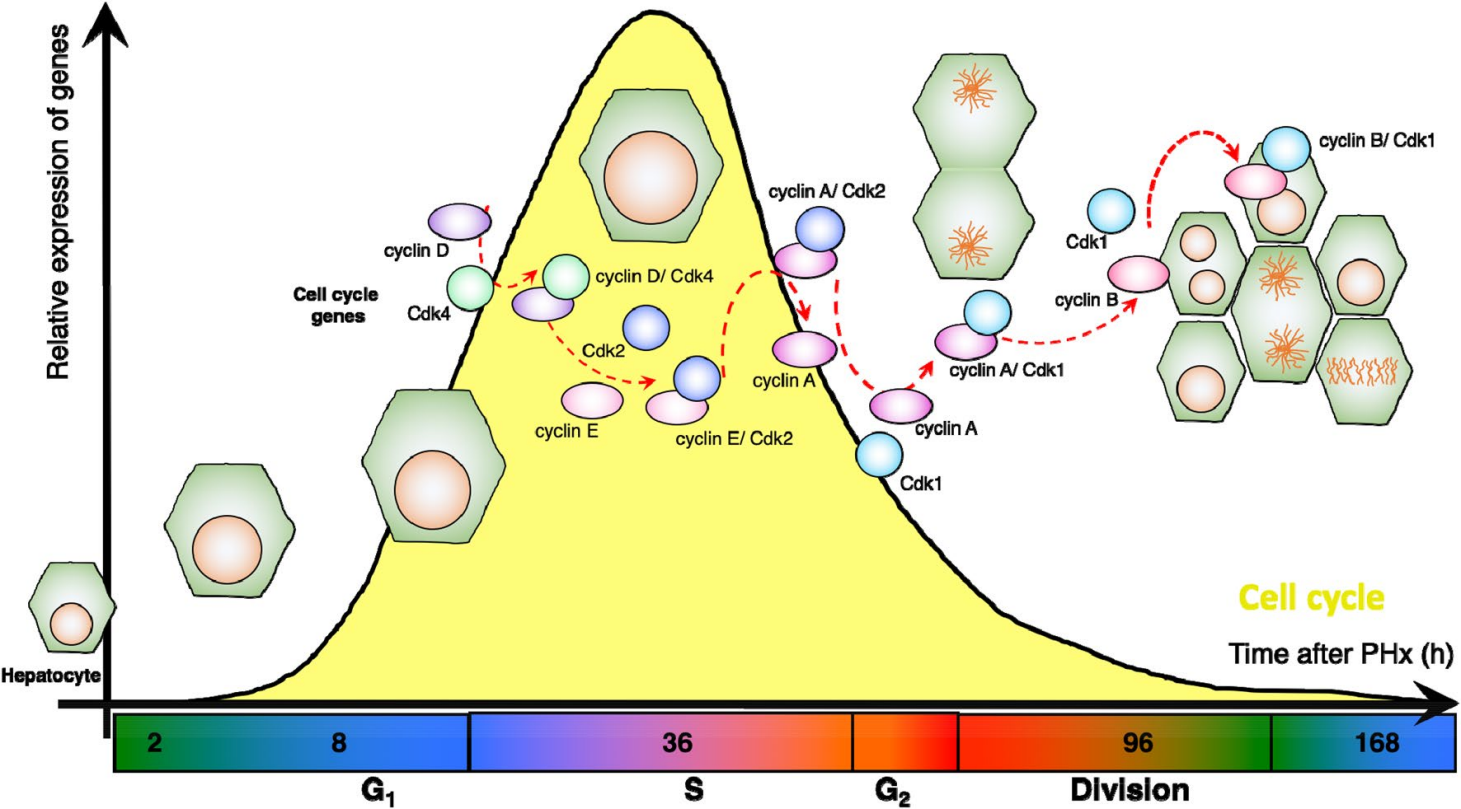
Regulation of cell cycle progression, cellular division and hypertrophy during regeneration. The progression phase of liver regeneration depends on the synchronized expression of cell cycle genes. Within hepatocytes, cyclins and CDKs, as well as their inhibitors, show a similar expression pattern to that observed in vitro models of cell culture. However, many new functions have been shown recently, such as regulation of DNA replication by CDK1, or interactions with metabolic pathways

During liver regeneration, hepatocytes display an equivalent pattern of expression of cell cycle genes comparable to that observed in cell culture models in vitro (for an exhaustive review on cell cycle regulators refer to Refs. [27, 28] and Fig. 2). For example, before liver resection, most genes involved in cell division like *Cdk1* and *cyclin B* are downregulated. Instead genes involved in quiescence like *p21*^*Cip1/Waf1*^ and *p27*^*Kip1*^ are highly expressed [12, 29–31]. During the initiation phase of liver regeneration, the expression of genes involved in G1 progression like *cyclin D* and *Cdk4* is induced [29]. Finally, during the progression phase of regeneration most cell cycle genes are actively expressed [32, 33] (Fig. 3). However, there are several exceptions to this rule. For example, the murine *cyclin B* transcript is regulated at different time points during liver regeneration, with a peak of expression that coincides with the peak of DNA replication (S-phase, ≈ 36 h after resection) [34, 35]. Additionally, in regenerating livers *cyclin A* and *cyclin B* are observed in the cytoplasmic, nuclear, and membrane containing fractions [36, 37]. In the same way, *Cdk1* is expressed as early as 30 h after hepatectomy, which is the period of DNA replication in regenerating hepatocytes [38]. These observations suggest that *Cdk1* may display novel functions outside of mitosis, which is supported by recent data [26, 38] but was not observed in vitro models. Collectively, these results suggest that *Cdk1* and its partners *cyclin A* and *cyclin B*, may possess non-canonical functions in the liver, different to the ones described in cell culture systems. We and others have shown that during liver regeneration *Cdk1* controls cell size and cell division [39], cell metabolism [26], and DNA replication [38, 40]. However, little data is available on the partners *cyclin A* and *cyclin B*. Hence, further studies may be essential to understand the roles of these cyclins during liver regeneration.

### Metabolic regulation during liver regeneration after acute liver damage

The liver is a vital center for homeostasis of glucose, lipid, and overall metabolism [41]. Therefore, acute tissue damage imposes significant stress to the general metabolic performance of the whole organ. Nevertheless, during liver regeneration after 2/3 liver resection, hepatocytes are able to remodel their metabolic network to promote regeneration while maintaining overall systemic metabolic balance. These early observations suggest that liver metabolism is robust. In this sense, metabolic pathways compensate for the loss of many individual enzymes, which is supported by mouse genetics [42–45]. However, liver specific deletion of some enzymes including nicotinamide phosphoribosyl transferase (*Nampt*) and several members of the P450 cytochrome reductase family leads to lethality after partial hepatectomy [46–48].

The changes in metabolic demands usually induce small rearrangements in metabolism through local mechanisms without substantial rewiring of the overall metabolic network [41]. However, 2/3 partial hepatectomy leads to both loss of tissue mass and glycogen, a major energy reservoir for systemic glucose and energy metabolism [49]. Major resection of glycogen in the liver, leads to a substantial reduction in blood glucose. This is particularly apparent during the initiation phase of liver regeneration when there is a significant increase in the oxidative status as a response to a whole body induction of catabolism [49]. Because glucose supply via glycogenolysis is limited, the energy required to promote liver regeneration may be partly acquired from alternative sources that are actively transported into hepatocyte, such as lipids [50]. In fact, deletion of the *Caveolin1 (Cav1)* gene induced hepatic failure during early phases of liver regeneration, indicating that lipid import into hepatocytes is essential to drive regeneration [51]. CAV1 is essential to transport lipids into hepatocytes. Therefore, these observations are in agreement with temporary hepatic steatosis being a key feature of the early stages of liver regeneration [52, 53]. Interestingly, physiological steatosis does not overlap with cell division during liver regeneration, which may imply that lipid metabolism and cell division are strictly temporally separated. However, these ideas need to be further tested.

**Fig. 4:**
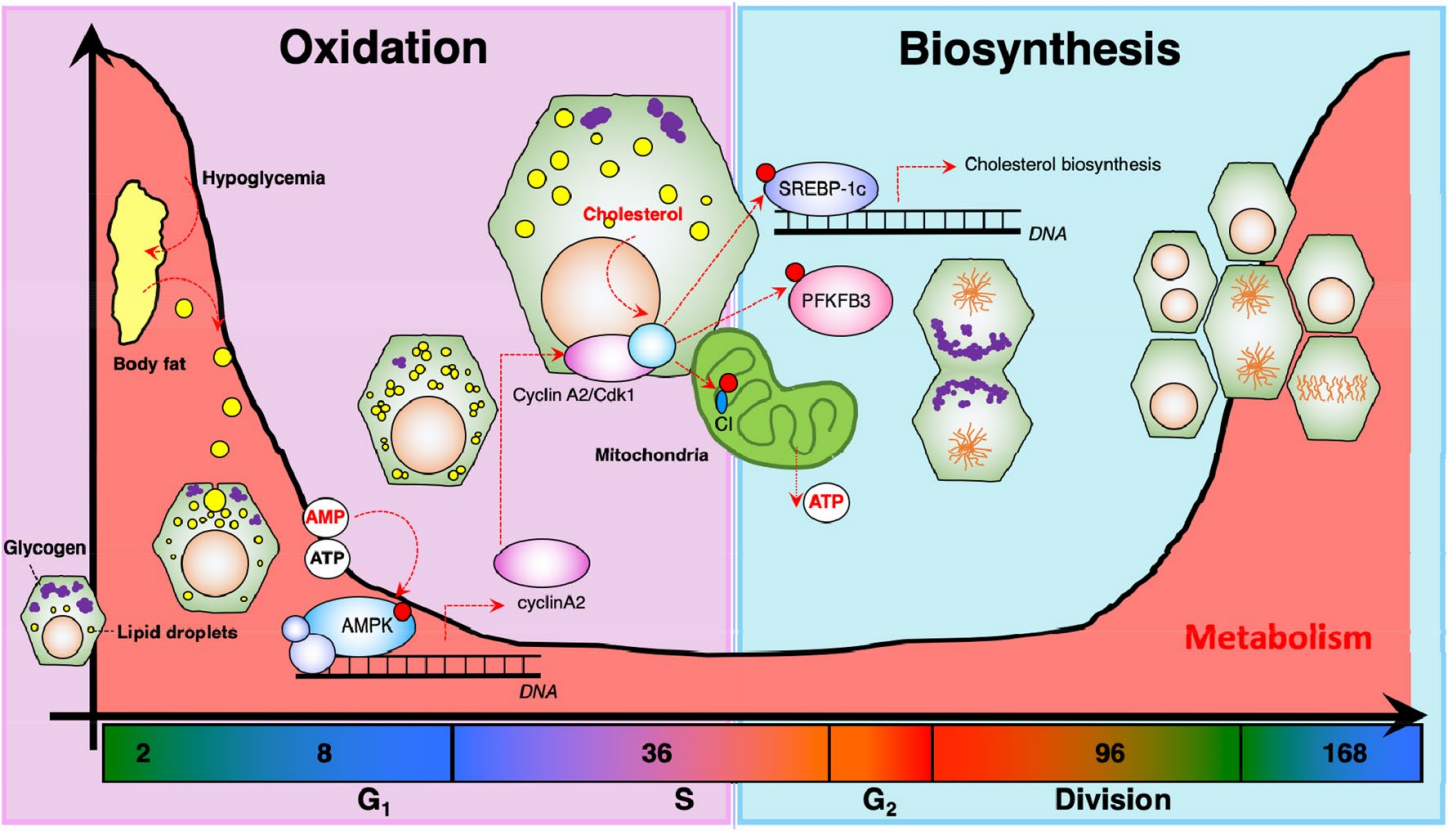
Interaction of cell cycle genes with major metabolic pathways. After 2/3 mass removal, there are significant changes in hemodynamic pressure in the liver, leading to oxidative stress. Moreover, resection of 70% of the hepatic glycogen stores leads to hypoglycemia, which in turn promotes lipolysis of adipose tissue. These lipids are transported into hepatocytes in the initiation phase of regeneration and produce energy through oxidation. When DNA replication and cellular division starts, there is a reduction of oxidation and concomitantly activation of biosynthetic pathways. In this context, CDK1 may regulate metabolite production in the mitochondria, like NADH and FAD, or it may directly phosphorylate transcription factors like SREBP-1c, or other enzymes involved in anabolic pathways like PFKFB3

During the termination phase of liver regeneration, hepatic gluconeogenic functions regain homeostatic levels, marked by a sharp increase in expression of *Akt* [44]. These events are followed by the activation of biosynthetic pathways to promote tissue homeostasis, accumulation of glycogen as well as normal glucose and lipid metabolic processes. Based on the main two primary sources of energy, we would like to propose a division of metabolic-related events during liver regeneration in two phases (1) oxidative and (2) biosynthetic (Fig. 4). The oxidation phase is distinguished by oxidative metabolism correlated to increases in cell size. This would last from the early minutes after liver resection to 30 h, when hepatic DNA synthesis starts to peak (Fig. 4). After that, the biosynthetic pathways will be active until metabolic homeostasis is reached.

### Interactions between cell cycle regulators and metabolism during acute liver damage

Reduction in blood glucose represents one of the primary metabolic markers of hepatic regeneration [26]. The characteristic short-term hypoglycemia is required for adequate liver regeneration. This is supported by clinical data indicating that patients with hyperglycemia typically experience an adverse regenerative outcome [54]. Another cause for decreased concentration of glucose in serum is the reduced expression of metabolic enzymes during the priming and progression phases [17]. We have recently published similar findings in mice employing a combination of transcriptomic, metabolomic and advanced molecular imaging [26].

During cellular division CDK1 phosphorylates specific subunits of the mitochondrial oxidative phosphorylation complex I [55, 56]. In this sense, CDK1 may couple cell division and energy metabolism. In post-mitotic tissues, CDK1 regulates gluconeogenic pathways by phosphorylation of FOXO-1 [57] and glycolytic genes like PFKFB3 [58, 59]. Following this line of evidence, we published that ablation of CDK1 in hepatocytes led to a significant reduction in NADH concentration because mitochondrial function is suboptimal [60]. These observations are in agreement with recent reports suggesting that deletion of *Nampt* reduced hepatocyte proliferation during regeneration [47]. Collectively, these results suggest an interplay between the oxidative state of hepatocytes and CDK1. Deletion of *Cdk1* in the liver led to a significant reduction in mitochondrial metabolic processes during S-phase [26]. A standard explanation for this is that DNA replication and oxidative metabolism are not compatible. Reactive oxygen species (ROS) generated as a side-product of oxidative metabolism would be detrimental when the maintenance of DNA fidelity is most critical [61, 62].

### Cholesterol and the cell cycle

In the previous sections, we highlighted that hepatic accumulation of lipid droplets is associated with the initiation of regeneration and not cellular division. Therefore, we speculate that the presence of either lipid droplets or their components are incompatible with proliferation. A lipid droplet is composed by triglycerides, lipoproteins, cholesterol and other components [63]. Cholesterol represents the fundamental structural component and thus is essential for its formation. In mammals there are two main sources of cholesterol, (1) gastrointestinal digestion and (2) biosynthesis. Regardless of the source, cholesterol is stored and metabolized as esterified cholesterol in pericentral hepatocytes [64]. As mentioned earlier, during liver regeneration the metabolic capacity of hepatocytes is heavily reduced, and it is expected that cholesterol production may be hindered as well. In fact, our preliminary data show that concentration of serum total, free and esterified cholesterol are significantly reduced during liver regeneration (unpublished).

In mice, hepatic *Srebp-1c* is an essential gene for the transcriptional activation of enzymes involved in cholesterol metabolic processes. Deletion of Srebp-1c in hepatocytes led to increased accumulation of cholesterol [65], with no changes in the composition of other lipids. When mice without *Srebp-1c* were subjected to 2/3 partial hepatectomy, hepatocyte division was significantly enhanced in comparison to wild type mice [65]. This was in agreement with previous observations that showed reduced expression of CDK1 and less mitotic cells upon cholesterol starvation [66, 67]. Together, these studies further support the idea that there might be a relationship between cholesterol metabolism and cellular division. We have shown that reduced division rate of cells during liver regeneration, led to a significant reduction in expression of enzymes from cholesterol and mevalonate pathway, suggesting an interaction between the machinery that regulates cell cycle and that of cholesterol synthesis. This is further supported by experiments indicating that during cell cycle progression, cells regulate their lipid composition and localization [68]. We are currently dissecting the molecular mechanism by which CDK1 may regulate cholesterol synthesis in hepatocytes. Forthcoming work should address the association between the formation of lipid droplets during physiological steatosis and cellular division during liver regeneration independently.

## The bidirectional interaction of metabolism and cell division during liver regeneration after chronic damage

NAFLD is characterized by a sustained insult to the parenchyma, with alternating cycles of damage and regeneration. The liver does not regenerate optimally in patients with NAFLD, NASH, and/or obese patients [69, 70]. Indeed, it has been proposed that steatosis in patients before liver resection is a risk factor for liver failure after hepatectomy [70] and causes postoperative complications [71]. Growing evidence has indicated that NAFLD is typically associated with increased synthesis of lipids derived from long-chain fatty acids, like palmitic and linoleic acids [72]. These macromolecules promote the characteristic inflammatory response through the production of lipotoxic molecules [72]. However, and more importantly, these bioactive lipid precursors usually activate lipid peroxidation, with a concomitant reduction in mitochondria function and increased oxidative stress by production of ROS [72]. In this context, cell cycle progression is impaired, with concomitant activation of apoptosis which further promotes inflammation and fibrosis. In contrast to a healthy liver, hepatocyte-dependent regeneration is diminished in the chronically damaged liver. In this section, the potential associations between cell division and metabolism will be discussed in two in models of diabetes, obesity, NAFLD, and NASH.

### Hepatocyte cell cycle progression in genetic models of obesity and diabetes

NAFLD evolves from the response of altered (1) lipid transport into hepatocytes; (2) increased production of lipids from hepatocytes; as well as (3) reduced export and consumption of lipids [63]. In diabetes and obesity, NAFLD is accompanied by changes in insulin metabolism [5]. In hepatocytes, insulin promotes the biosynthesis of lipids by inhibiting fatty acid oxidation, with leptin being essential for the insulin response [73]. Murine models with mutations in either leptin or the leptin receptor gene led to body fat accumulation, and the onset of obesity and diabetes mellitus. Initial observations using these mouse models demonstrated impaired cell cycle progression of hepatocytes after liver regeneration. For example, hepatocytes from Zucker rats arrested in G1, with a concomitant lower expression of cyclin D1 after hepatectomy [74]. These initial results led to the hypothesis that accumulation of fat droplets may negatively regulate hepatic cell division and reduce cell proliferation. Besides, in *ob/ob* mice survival rate after liver resection was significantly also lower than in wild type. These mice do not produce leptin, and displayed significant hypoglycemia as well as impaired hepatocellular proliferation after liver resection [75]. These effects occur with no difference in serum concentration of pro-proliferative hormones during liver regeneration like TNFα, IL-6, and insulin. In a third model, *fa/fa* rats displayed elevated concentration of leptin in blood and a similar reduction in the survival after hepatectomy [76]. Nonetheless, leptin replacement restored TNFα and IL-6 release and induced *cyclin D1*, suggesting that leptin may play a central role in the interaction between cell cycle regulators and lipid metabolism [77]. A conflicting result arises from the fact that leptin supplementation improved hepatic division, but did not reduce the onset of liver failure [78, 79]. In addition, intraperitoneal injection of leptin in wild type mice with no obesity increased mitotic counts during liver regeneration [78]. Collectively, these results suggest that adipokines play a significant role in hepatocellular proliferation. Similar results were observed in a model of fatty liver in ALPPS rats [80]. In summary, leptin is essential for hepatic division and together with other factors may act in recovery after hepatectomy [81]. Subsequent research should address the role of adipokines like leptin in regulation of hepatic cell cycle genes.

### Cell cycle progression in models of diet manipulation

**Fig. 5:**
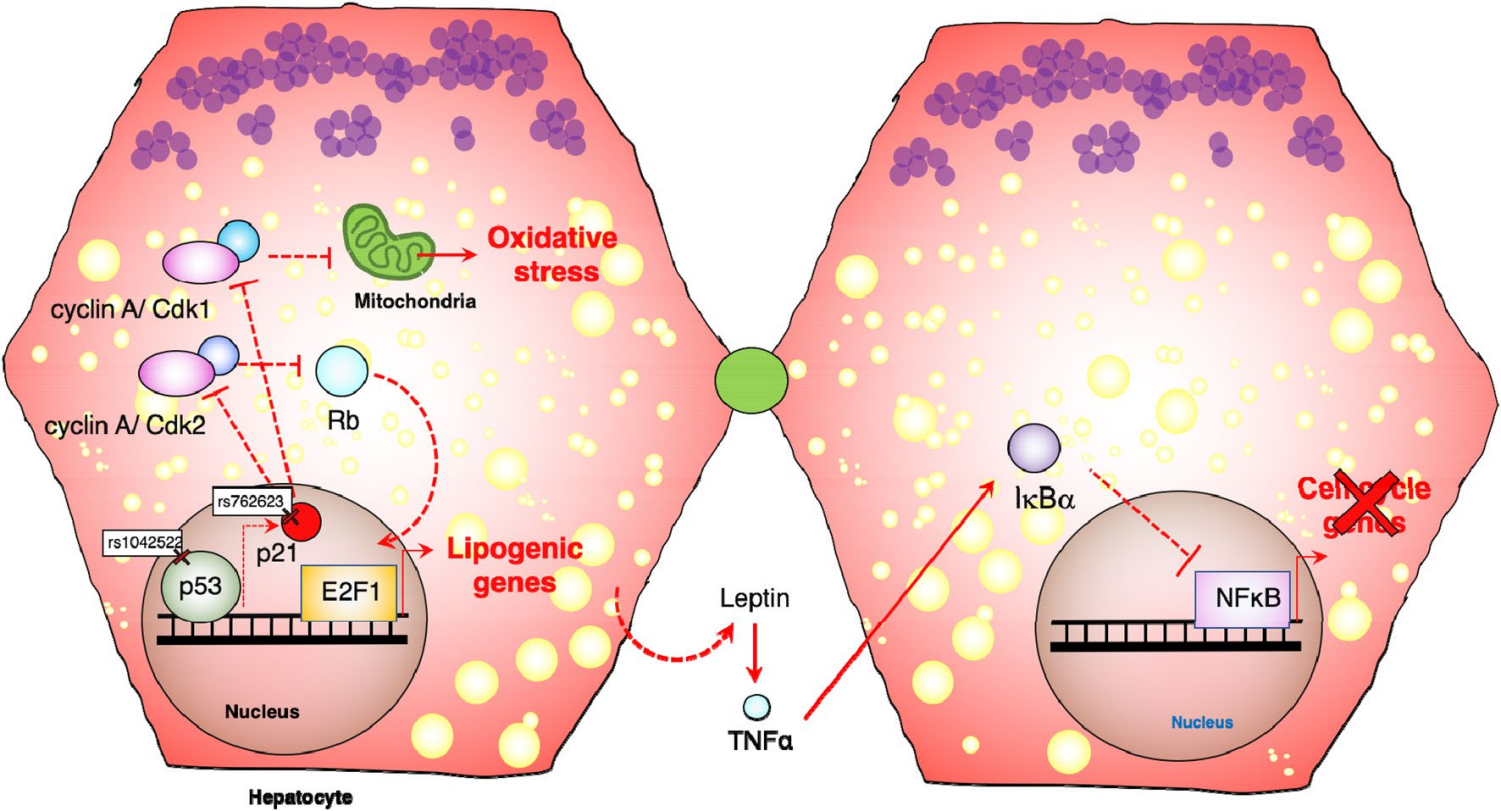
Bidirectional regulation of cell cycle and metabolism in NAFLD. Development of NAFLD depends on daily habits, diabetes, obesity, genetic predispositions and/or other cooperating factors. Recently, a common SNP in p53 in NAFLD patients hinted that hyperactivation of this transcription factor may promote fatty liver disease. The proposed involves increases in the expression of p21. Alternatively, other SNPs have been found in p21 that lead to its overexpression in NAFLD. Importantly, p21 may predispose to steatohepatitis by two mechanisms that may or may not work together. Initially, by increasing phosphorylation of the retinoblastoma protein (RB), it may promote constant activation of the transcription factor E2F1 leading to increase in lipogenesis. Similarly, blocking functions of CDK1 may lead to increases in oxidative stress which promotes NAFLD due to chronic damage

To promote lipid overloading independently of a genetic mutation, many experiments provide mice with a high-fat diet (HFD). These models are more clinically-relevant compared to mice with mutations in enzymes. HFD leads to high calorie intake, body weight increase, liver steatosis and reduced regeneration caused by metabolic surplus [82, 83]. Liver regeneration in mice fed with HFD was accompanied by increased TNF-α potentially amplified by leptin which promoted increased levels of IκBα preventing NF-κB activation. Consequently, expression of NF-κB target genes, including *cyclin D1* was reduced [82] (Fig. 5) leading to a decrease in proliferation of hepatocytes. Mice fed with HFD display a delayed incorporation of BrdU after surgery [84], indicating that steatosis not only inhibits cell division but also delays DNA replication. Expression of *cyclin D1* and *cyclin E2* are usually higher before partial hepatectomy in mice fed with HFD. However, there was no significant difference in expression between mice fed with HFD and Chow diets during liver regeneration [84]. In contrast, *cyclin A2* and *cyclin B1* are significantly reduced during liver regeneration in mice fed with HFD, further suggesting that cell division might be impaired [82]. In conclusion, these observations suggest that leptin-related steatosis due to HFD may regulate cell cycle progression differentially by downstream control of the inflammatory pathway NF-κB, which is supported by studies done in mice with lipodystrophy [85]. Lipid synthesis and degradation, as well as the correct localization of the corresponding enzymes are essential for cell division [68]. To the same extent, cell cycle effectors regulate directly or indirectly lipid synthesis in hepatocytes. To follow up on these topics, in the next section we will summarize current data on how cell cycle regulators control lipid synthesis. We will particularly focus on a novel axis of molecular signaling that we describe as the p21-CDK1-E2F1-lipid synthesis axis.

## Lipid metabolism and cell cycle in NAFLD

NAFLD pathogenesis displays a broad phenotype ranging from cellular steatosis, characterized by micro and macrovesicles, to NASH with substantial inflammation and fibrosis. As mentioned above, an increasing amount of evidence indicates that the synthesis of a particular group of lipids in NAFLD hepatocytes may initiate the activation of inflammation. In recent years, several reports have suggested that the direct consequence of the chronic metabolic inflammation is the activation of senescence-associated secretory phenotype (SASP) [4] with a concomitant increase in expression of the senescence marker *p21*^*Cip1/Waf1*^ [86]. Cellular senescence is a biological process activated by an irreversible cell cycle arrest. Replicative senescence is associated with aging of tissues, mainly caused by shortening of the telomeres. In contrast, stress-induced premature senescence is caused by DNA damage caused by intra or extracellular factors such as oxidative stress. Either type of senescence leads to a DNA damage response, which inhibits cell cycle progression. In NAFLD, chronic metabolic surplus generates many of the molecules involved in cellular senescence, like ROS, with a concomitant change in gene expression. Several genome wide association studies (GWAS) have found a significant correlation between increased frequency of single-nucleotide polymorphisms (SNPs) in the *Cdkn1a* gene (encoding p21^Cip1/Waf1^) that leads to increased p21^Cip1/Waf1^ expression in NAFLD [86, 87]. Increases in expression of p21^Cip1/Waf1^ is one of the main markers of senescence [87], because it is a potent negative regulator of CDK1 activity. Moreover, our data demonstrates that long term deletion of *Cdk1* in hepatic cells induced hepatic expression of p21^Cip1/Waf1^, cellular senescence, fibrosis and inflammation (unpublished data). In this section we will summarize the current role of hepatic cell cycle regulators in lipid biosynthesis, senescence and onset of NAFLD.

### The p21-Cdk1-E2F1 axis

Recent findings have indicated that NAFLD livers display lower expression of *Cdk1* [88]. CDK1 is essential for mitotic division, but in hepatic cells CDK1 may have other functions beyond mitosis. For example, we have shown that deletion of *Cdk1* in hepatocytes leads to increases in nuclear and cell size [21]. DNA re-replication by endoreduplication may be the underlying cause of this effect. During endoreduplication, hepatic cells may generate oxidative stress leading to chronic liver damage [88]. In this setting, p21^Cip1/Waf1^ negatively regulates CDK1, and therefore increased levels of p21^Cip1/Waf1^ in liver disease may correlate with reduced activity of CDK1 [88]. Therefore, it may be therapeutically feasible to reduce the expression of p21^Cip1/Waf1^ in hepatocytes to reduce NAFLD. However, the pleiotropic functions of p21^Cip1/Waf1^ are likely to make it an “undruggable” target. Interestingly, it was recently shown that senotherapeutic drugs improved liver regeneration when p21 was overexpressed further suggesting that SASP may inhibit liver regeneration [89].

An important function for p21^Cip1/Waf1^ is the transcriptional regulation of cell cycle genes. Typically, p21^Cip1/Waf1^ binds with high affinity to and inhibits CDK2/cyclin E activity [39] which leads to hypo-phosphorylation of the RB protein [90]. Hypophosphorylated RB remains bound to E2F1, repressing its activation and cell cycle progression. Recent results highlighted a number of novel metabolic functions of E2F1 [91]. For example, E2F1 mRNA and protein are increased in NASH livers [92]. On top of that, hepatic deletion of *E2f1* significantly decreased fibrosis as a consequence of downregulation of the *Egr-1* gene [93, 94]. Moreover, E2F1 regulates the expression of essential genes in lipid synthesis such as *Acaca, Fasn, Scd1, Srebp1c,* and *Chrebp* [92]. The interaction of p21^Cip1/Waf1^ with CDK1 may lead to inactivation of E2F1. E2F1 also drives the expression of *Cdk1* [94]. In this fashion, p21^Cip1/Waf1^ regulates cell cycle progression (Fig. 5), cellular division, senescence and metabolism through the expression of numerous genes, including lipogenic enzymes {for review see [95]}.

Finally, the transcription of p21^Cip1/Waf1^ is activated by p53. In response to DNA damage, p53 promotes the expression of p21^Cip1/Waf1^, leading to cell cycle arrest and senescence [22, 96, 97]. Simultaneously, p53 regulates the transcription of many genes involved in glycolysis, lipogenesis and other metabolic pathways [98]. Particularly interesting is the discovery of frequent SNPs variants in p53 that predispose patients to develop obesity and NAFLD [99] (Fig. 5). When these variants were introduced in mice fed with HFD, they caused significant increases in body weight with liver steatosis, inflammation, and fibrosis [100]. These mice also displayed a remarkable increase in the expression of p21^Cip1/Waf1^ and inflammatory genes. Moreover, under fasting conditions, hepatic cells accumulate lipid droplets which correlate with increased expression of both p53 [99, 100] and p21 [86, 87]. Collectively, these observations suggest that p53 may fulfill essential roles upstream of p21 ^Cip1/Waf1^ and Cdk1 in NAFLD.

## Conclusion and future perspective

The regulation of cell cycle progression, its components and the interactions among them has been meticulously studied for more than four decades. Most of the basic functions discovered in vitro have been validated in vivo with similar physiological significance. However, an increasing number of studies now indicate that a large number of cell cycle regulators, including CDK1, influence metabolism which are new interactions that may not depend on cell cycle progression. This may not be surprising as cells need to integrate their growth with cell proliferation, into a wider network of biosynthetic pathways. However, this point has been largely ignored as the focus has been in the upstream signaling pathways regulating cell cycle progression. Studying the interplay between cell cycle and metabolism in detail will lead to development of more refined therapies for the treatment of diseases.

Particularly important is to understand the role of key cell cycle regulators CDK1 and p21 in regulating metabolic pathways during acute and chronic liver damage. The reasoning behind this is that if there is a distinction between the pro-regenerative functions of these two, and other functions, then it may be able to target them in NAFLD. It is well established that liver regeneration is dramatically reduced in patients with NAFLD, and therefore resection and transplantation is not an ideal therapy for this group of patients. This is exacerbated in the more advanced stage of disease, like NASH. In this setting, targeting pro-regenerative pathways of hepatocytes may lead to a better outcome. Our group has actively studied the non-canonical functions of Cdk1, but those of p21 have been neglected. For example, we have preliminary data indicating that downregulation of Cdk1 may lead to a NAFLD-like phenotype in the long term, with fibrosis and steatosis. Thus, in NAFLD patients it would be therapeutically relevant to disrupt the p21-Cdk1 axis, with increasing Cdk1 functions while reducing those of p21. However, there is a gap on our understanding of the functions of increased expression of p21 and its activity in the context of deletion of *Cdk1*, or if the observed phenotype is a Cdk1-autonomous effect.

Finally, studies of NAFLD have focused mostly on targeting inflammatory or non- parenchymal cells. Thus, studying the cell cycle and non-canonical functions of p21 and Cdk1 in hepatocytes may lead to far reaching therapeutic approaches for either early NAFLD or specially advanced NASH. Ideally personalized therapies may be developed, in which expression of p21 would be reduced in patients with SNPs in the promoter region. Following this approach, we will be able to reduce the signaling associated with increased p21 activity. These future discoveries will have direct implications in the development of therapeutic agents targeting both, metabolism and cell division, for these common liver diseases, NAFLD and NASH.
